# Identification of Terpene Compositions in the Leaves and Inflorescences of Hybrid *Cannabis* Species Using Headspace-Gas Chromatography/Mass Spectrometry

**DOI:** 10.3390/molecules28248082

**Published:** 2023-12-14

**Authors:** Sangin Lee, Eun Jae Kim, Eunjeong Kwon, Seo Jeong Oh, Mansoo Cho, Chul Min Kim, Wonwoong Lee, Jongki Hong

**Affiliations:** 1 College of Pharmacy, Kyung Hee University, Seoul 02447, Republic of Korea; ahfqqhsi@naver.com (S.L.); dmswo3537@naver.com (E.J.K.);; 2 Graduate School of Techno Design, Kookmin University, Seoul 02707, Republic of Korea; chomansoo@gmail.com; 3 Department of Horticulture Industry, Wonkwang University, Iksan 54538, Republic of Korea; chulmin21@wku.ac.kr; 4 Research Institute of Pharmaceutical Sciences, College of Pharmacy, Woosuk University, Wanju 55338, Republic of Korea

**Keywords:** hybrid *Cannabis* species, terpenes, leaf, inflorescence, headspace, gas chromatography, mass spectrometry, statistical analysis

## Abstract

Although cannabidiol and tetrahydrocannabinol in *Cannabis* species exert their pharmacological effects via the endocannabinoid system, it is believed that other phytochemicals, particularly terpenes, can modulate therapeutic outcomes through the entourage effect. Therefore, to gain a better understanding of the pharmacological effects of *Cannabis*, obtaining information on phytochemical compositions, including mono-, di-, and sesqui-terpenes in *Cannabis* species is essential. Applying a sophisticated analytical method is indispensable. In this study, headspace-gas chromatography/mass spectrometry (HS-GC/MS) was employed to identify major terpenes in the leaves and inflorescences of hybrid *Cannabis* species. The incubation time and temperature conditions for HS-GC/MS were optimized. This method was successfully applied to the leaves (*n* = 9) and inflorescences (*n* = 7) of hybrid *Cannabis* species. A total of 26 terpenes in *Cannabis* species were detected, and six major components, such as α-pinene (9.8–2270 μg/g), β-pinene (2.6–930 μg/g), myrcene (0.7–17,400 μg/g), limonene (1.3–300 μg/g), β-caryophyllene (60–3300 μg/g), and α-humulene (40–870 μg/g), were quantified. Each sample showed different terpene compositions, but six major terpenes among all the terpenes detected were consistently found in both the leaves and inflorescences of hybrid *Cannabis* species. In this study, the six major terpenes’ potential in hybrid *Cannabis* species was evaluated as biomarkers to distinguish hybrid *Cannabis* species samples. This study contributes to a better understanding of the entourage effect of *Cannabis*-based botanical drugs.

## 1. Introduction

*Cannabis* species contain various bioactive phytochemicals, categorized as cannabinoids and non-cannabinoids [1], used for food, medicine, and even ornamental plants [2]. Their unique pharmacological effects have generated increased interest in many areas, including academia, industry, and the government. Among the bioactive phytochemicals in *Cannabis*, tetrahydrocannabinol (THC) and cannabidiol (CBD) have psychoactive effects by binding to endocannabinoid receptors [3] and therapeutic effects for epilepsy, pain, and drug addiction [4]. Although both THC and CBD are known to be the most potent bioactive compounds, other phytochemicals in *Cannabis* species also exhibit characteristic effects on the human body [5]. In particular, terpenes reportedly contribute to the entourage effect, which can modulate the unique psychoactive effects of cannabinoids such as THC and CBD [6]. Therefore, to better understand the entourage effect, identifying and quantifying not only cannabinoids but also terpenes in *Cannabis* species is crucial.

To enhance their psychoactive and therapeutic effects, crossbreeding different *Cannabis* species has become an industrial and commercial preference rather than cultivating original *Cannabis* species. Consequently, in the *Cannabis* industry and market, finding landrace *Cannabis* that is not hybrid *Cannabis* is challenging [7]. Hybrid *Cannabis* species are known to have distinct effects and are promoted and sold based on their unique entourage effects. Although cannabinoid contents (THC and CBD) in individual hybrid *Cannabis* species have been presented, providing the terpene contents in these strains to understand the characteristic entourage effect of individual hybrid *Cannabis* is essential.

Numerous analytical methods have been developed to determine terpenes in *Cannabis* plants [8,9,10,11,12]. Although high-performance liquid chromatography (HPLC) has been used to determine various bioactive phytochemicals in *Cannabis* species [13,14,15], gas chromatography (GC) has been widely employed to analyze terpenes contributing to the fragrance and flavor of products [16]. In particular, flame ionization detection (FID) realizes a simple and easy operation method combined with GC, while mass spectrometry (MS) provides reliable qualification and quantification results [17]. To analyze volatile terpenes in *Cannabis* plants, a delicate sample preparation method should be performed to extract volatile terpenes without significant losses. Solid-phase microextraction (SPME) has been widely used to extract terpenes from natural products [18,19] since it is one of the representative methods to extract volatile compounds from various matrices. Nonetheless, optimization procedures for SPME conditions (such as temperature, solvents, and fibers) should precede sample application and be accompanied by intensive labor and time [20]. A headspace (HS)-SPME method may serve as an alternate simple sample preparation method with automated operation and no solvent usage [21]. Another excellent alternative might be an automated HS method, allowing for direct extraction of volatile compounds from various matrices without needing fibers and solvents [22]. Consequently, automated HS methods have been widely employed to extract bioactive volatile phytochemicals from plant samples [11,23,24].

In this study, we developed an HS-GC/MS method to determine volatile terpenes in the leaves (*n* = 9) and inflorescences (*n* = 7) of hybrid *Cannabis*. Twenty-six terpenes in hybrid *Cannabis* samples were detected and identified using this method. The developed HS-GC/MS method was optimized and validated to quantify six major and abundant terpenes, including α- and β-pinene, myrcene, limonene, β-caryophyllene, and α-humulene, in the leaves and inflorescences of hybrid *Cannabis* species. Since individual *Cannabis* samples exhibited characteristic distributions for terpenes, even in leaves or inflorescences, they could not be categorized into similar distributions. In conclusion, this study provides characteristic terpene distributions and quantification results for six major terpenes in the leaves and inflorescences of hybrid *Cannabis*. Furthermore, this study helps to better understand the characteristic entourage effect of terpenes in individual hybrid *Cannabis*.

## 2. Results and Discussion

### 2.1. Optimization of HS-GC/MS Conditions

Although GC/FID provides several advantages, such as ease, simplicity, and low cost, GC/MS has been the ‘gold standard’ for identifying and quantifying volatile phytochemicals in plant samples due to its indispensable sensitivity and selectivity [25]. Moreover, when combined with an automated HS system, a GC/MS method reduces the need for labor, minimizes processing time, and decreases the use of harmful solvents. Therefore, in this study, we employed an HS-GC/MS method to identify and quantify terpenes in the leaves and inflorescences of hybrid *Cannabis*.

**Figure 1:**
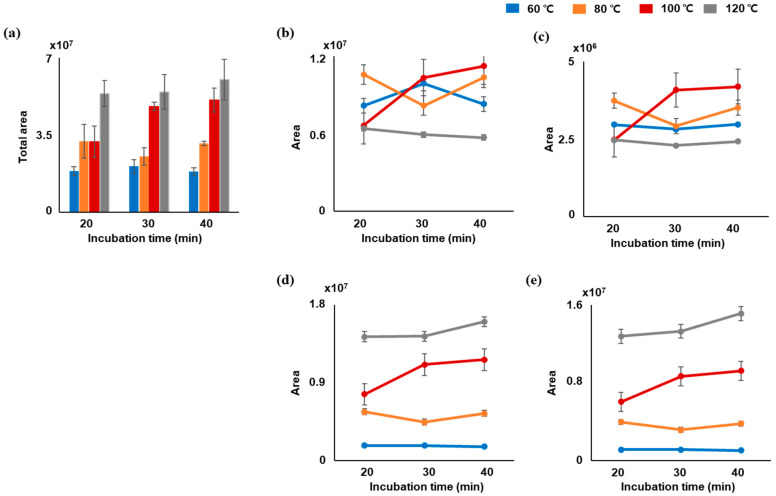
Influence of incubation time and headspace temperature on terpene extraction efficiency in leaf samples of Victory: (**a**) total peak area of terpenes and individual peak area of terpenes such as (**b**) α-pinene, (**c**) myrcene, (**d**) β-caryophyllene, and (**e**) α-humulene.

To efficiently extract terpenes from the leaf and inflorescence samples, the HS conditions were optimized in terms of incubation time and temperature using a standard solution of representative terpenes, including α-pinene, myrcene, β-caryophyllene, and α-humulene. As shown in Figure 1, the targeted terpenes were more affected by temperature than by incubation time. In particular, the most effective total area for terpene extraction was at 120 °C. However, it should be noted that terpenes with high volatility, such as α-pinene and myrcene, exhibited lower levels than other investigated temperatures. This finding might be attributed to the degradation of terpenes caused by high temperatures [26]. Although the incubation time parameter had a lesser influence on terpene extraction efficiency, an incubation time of 30 min at 100 °C was shown to be effective.

Furthermore, we preliminarily investigated total ion chromatograms of representative hybrid *Cannabis* leaf samples at varying incubation temperatures, where incubation was performed for 30 min. As shown in Figure S1, peak areas for all terpenes in leaf samples increased until 100 °C, while highly volatile terpenes (early eluted) were degraded at 120 °C. Moreover, signal noise, resulting from matrix influences, increased at 120 °C. Therefore, we selected 100 °C and 30 min as the optimal incubation temperature and time, respectively.

### 2.2. Investigation of Terpenes in the Leaves and Inflorescences

**Figure 2:**
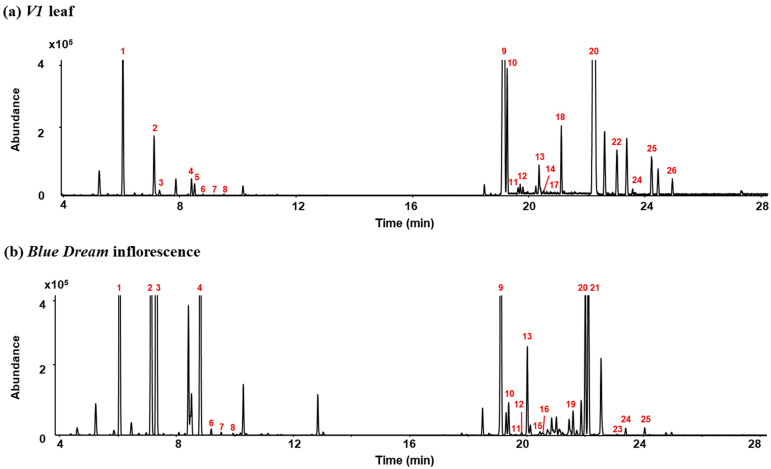
Total ion chromatograms for (**a**) V1 leaf and (**b**) Blue Dream inflorescence samples of hybrid Cannabis. (Peak identities are as follows: 1. α-Pinene, 2. β-Pinene, 3. Myrcene, 4. Limonene, 5. Eucalyptol, 6. E-β-Ocimene, 7. γ-Terpinene, 8. Z-Sabinene hydrate, 9. β-Caryophyllene, 10. α-Bergamotene, 11. α-Guaiene, 12. E-β-Farnesene, 13. α-Humulene, 14. Alloaromadrene, 15. β-Selinene, 16. α-Selinene, 17. Z,E-α-Farnesene, 18. β-Bisabolene, 19. β-sesquiphellandrene, 20. E-α-Bisabolene, 21. Selina-3,7(11)-diene, 22. Caryophyllene oxide, 23. Guaiol, 24. γ-Eudesmol, 25. Bulnesol, 26. α-Bisabolol).

The optimized HS-GC/MS method was preliminarily applied to collected leaf and inflorescence samples of hybrid *Cannabis*. As shown in Figure 2, a total of 26 terpenes were detected in leaf and inflorescence samples using HS-GC/MS. The detected terpenes were identified based on the National Institute of Standards and Technology (NIST) database and their mass spectral patterns. To further confirm the identified terpenes, the Kovats index (KI) was calculated using an alkane standard solution (C_8_–C_20_) and compared to reference KI [27]. The KI and characteristic ions for the 26 terpenes in hybrid *Cannabis* are summarized in Table 1.

To investigate the individual terpene compositions of leaf and inflorescence samples in hybrid *Cannabis* species, the relative abundance of all peaks were calculated based on the total ion chromatograms’ area under the curve. As shown in Table 2, several terpenes, including α- and β-pinenes, myrcene, limonene, β-caryophyllene, bergamotene, and α-humulene, were presented in all leaf samples of hybrid *Cannabis*. However, the guaiol terpene was not detected in all leaves, and bulnesol was only detected in the Blue Dream variety. The overall terpene compositions in inflorescence samples are shown in Table 3. As shown in Table 3, most terpenes were detected in all inflorescences of hybrid *Cannabis* species, except for the bulnesol terpene, which was only detected in Blue Dream, similar to the leaf samples. As shown in Table 2 and Table 3, even though some leaf and inflorescence samples originated from the same hybrid *Cannabis* species, including White Widow, individual terpene compositions substantially differed between leaves and inflorescences. To investigate the relationship between plant organs and/or hybrid *Cannabis* species, all hybrid *Cannabis* plants should be grown under uniform growing conditions since spatial differences, organs, and locations can influence individual terpene accumulation [28]. Among the identified 26 terpenes, six monoterpenes (including α- and β-pinenes, myrcene, limonene, β-caryophyllene, and α-humulene) were commonly detected in both the leaves and inflorescences of hybrid *Cannabis* species. These six terpenes were well-known as predominant terpenes [29] and may contribute to the “entourage effect” of cannabinoids [30].

**Figure 3:**
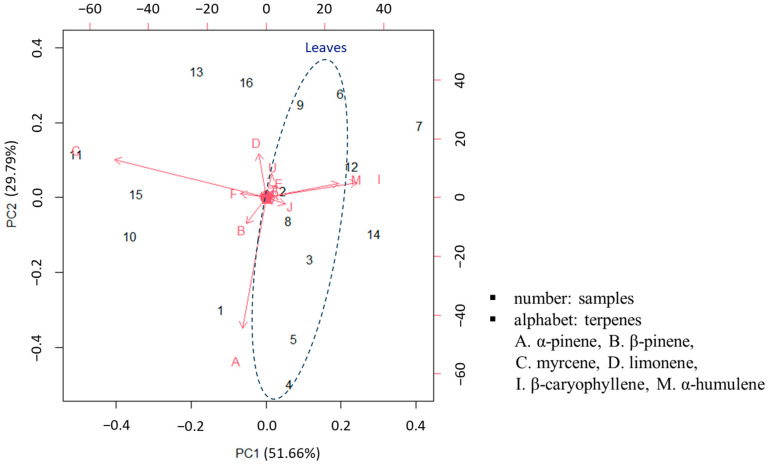
Principal component analysis (PCA) of hybrid *Cannabis* using relative abundances of peak areas for 26 terpenes. (Marks were identified as follows: 1. Cherry Blossom leaf, 2. V1 leaf, 3. V4 leaf, 4. White Widow leaf, 5. Chung Sam leaf, 6. Blue Dream leaf, 7. Bubble Gum leaf, 8. Purple leaf, 9. Victory leaf, 10. Cherry Blossom inflorescence, 11. V1 inflorescence, 12. V4 inflorescence, 13. White Widow inflorescence, 14. Chung Sam inflorescence, 15. Blue Dream inflorescence, 16. Bubble Gum inflorescence, A. α-Pinene, B. β-Pinene, C. Myrcene, D. Limonene, E. Eucalyptol, F. E-β-Ocimene, G. γ-Terpinene, H. Z-Sabinene hydrate I. β-Caryophyllene, J. α-Bergamotene, K. α-Guaiene, L. β-Farnesene (E), M. α-Humulene, N. Alloaromadrene, O. β-Selinene, P. α-Selinene, Q. Z,E-α-Farnesene, R. β-Bisabolene, S. β-sesquiphellandrene, T. E-α-Bisabolene, U. Selina-3,7(11)-diene, V. Caryophyllene oxide, W. Guaiol, X. γ-Eudesmol, Y. Bulnesol, Z. α-Bisabolol).

Based on the relative abundance of peak areas for terpenes, hierarchical cluster analysis (HCA) was performed to cluster organs (leaves and inflorescences) in hybrid *Cannabis* (Figure S2). Most leaf and inflorescence samples of hybrid *Cannabis* species could not be clustered by individual strains except for Bubble Gum. However, we speculated that the terpene compositions may be related to their organ types (leaves and inflorescences). Furthermore, principal component analysis (PCA) was also performed on the data set without scaling to find major terpenes, which can distinguish individual leaf and inflorescence samples of hybrid *Cannabis*. Based on PCA results, 51.66% and 29.79% of the variance was explained by PC1 and PC2, respectively. As shown in Figure 3, leaf samples could be grouped. Furthermore, samples from the leaves and inflorescences of White Widow and Blue Dream could be separated since individual leaves or inflorescences had characteristic terpene compositions, respectively. Six terpenes (α- and β-pinenes, myrcene, limonene, β-caryophyllene, and α-humulene) out of 26 terpenes have greater potential to identify individual leaves and inflorescence samples of hybrid *Cannabis* than other terpenes. These six terpenes would likely contribute to variance explanations for PC1 and PC2 since they were consistently and predominantly present in all leaves and inflorescences of hybrid *Cannabis*. Although several characteristic terpenes (such as guaiol found in Blue Dream inflorescences) may also be potential markers, they cannot separate all leaf and inflorescence samples of hybrid *Cannabis* species. Therefore, in this study, we quantified the six major and abundant terpenes as biomarkers using HS-GC/MS.

To quantify the six major terpenes in the leaves and inflorescences of hybrid *Cannabis* species, commercially available authentic terpene standards were employed. The HS-GC/MS method was validated in terms of quantification limits, calibration range, linearity, precision, and accuracy. Table S1 summarizes the overall data and validation results for quantifying six major terpenes using the HS-GC/MS method.

### 2.3. Quantification of the Six Major Terpenes in the Leaves and Inflorescences of Hybrid Cannabis Species

In this study, the validated HS-GC/MS method was employed to determine six major terpenes in the leaves (*n* = 9) and inflorescences (*n* = 7) of hybrid *Cannabis* species. As depicted in Table 4, quantified terpenes were found to be highly presented in the inflorescence sample of Cherry Blossom compared to other hybrid *Cannabis*. Furthermore, the quantification results for most terpenes in inflorescences were higher than in leaves. In the inflorescences of White Widow, Chung Sam, and V4, β-caryophyllene content was the most abundant. The biochemical diversity of terpenes in *Cannabis* makes it challenging to predict the pharmacological “entourage effect” of *Cannabis*. Therefore, the quantification results of the six terpenes with characteristic bioactive effects may help demonstrate their distinctive therapeutic outcomes and the “entourage effect” of individual leaf and inflorescence samples of hybrid *Cannabis*. For example, since α-pinene has antioxidative and anti-inflammatory effects [31,32,33], the inflorescence of Cherry Blossom may be more effective at relieving pain when combined with cannabidiol in *Cannabis* [34]. Furthermore, due to the analgesic and anti-cancer effects of β-caryophyllene [35], several hybrid *Cannabis*, including inflorescences of White Widow, Chung Sam, V4, and Cherry Blossom and leaves of Cherry Blossom and Victory, may be more suitable for cancer patients. Since both myrcene and limonene are flavor and fragrance chemicals, the inflorescences of Cherry Blossom, V1, White Widow, and Bubble Gum may be widely preferred by *Cannabis* users for their potential to inhibit *Cannabis* use disorders, including vomiting and nausea. Table 4 summarizes the overall calculated quantification results for the six terpenes in all leaves and inflorescences of hybrid *Cannabis*.

## 3. Experimental

### 3.1. Chemicals and Materials

Analytical grade methanol (MeOH) and ethyl acetate (EA) were purchased from J. T. Baker (Phillipsburg, NJ, USA). Deionized water (DW) was obtained using a Milli-Q purification system (Millipore Co., Bedford, MA, USA). Analytical grade standards of α- and β-pinene, myrcene, limonene, eucalyptol, β-caryophyllene, α-humulene, and alkane standard solutions (C_8_–C_20_) were purchased from Sigma–Aldrich (St. Louis, MO, USA). For internal standards, nonane and tetradecane were also purchased from Sigma–Aldrich (St. Louis, MO, USA). Leaves (*n* = 9) and inflorescences (*n* = 7) of hybrid *Cannabis* species were provided by Nongboo Mind (Seoul, Republic of Korea). All *Cannabis* samples were hybrid *Cannabis* species (combinations of indica and sativa) strictly supervised by the Korean Government. Therefore, only a limited number of *Cannabis* samples were allowed to be investigated in this study. All collected samples were sealed and stored in a freezer at −20 °C until analysis.

### 3.2. Sample Preparation

Fresh leaves and inflorescences from hybrid *Cannabis* species were prepared by removing superficial moisture with natural drying at room temperature, chopped using scissors, and weighed at 50 mg using an analytical balance (Mettler Toledo, Columbus, OH, USA). Weighed leaf and inflorescence samples were transferred into 10 mL headspace vials.

### 3.3. HS-GC/MS Conditions

To optimize headspace conditions, the incubation temperature (60–120 °C) and time (20–40 min) were tested. The sample was incubated at 100 °C for 30 min. The syringe temperature, fill speed, and injection speed of the automated headspace were 120 °C, 100 μL/s, and 500 μL/s, respectively. GC-MS analysis was performed by an Agilent 6890N gas chromatograph combined with an Agilent-5973 mass spectrometer equipped with electron ionization (EI) and a quadrupole analyzer. Separation was achieved using an Agilent Technologies DB-5MS column (30 m × 0.25 mm i.d., film thickness of 0.25 µm, J&W Scientific, Folsom, CA, USA). The sample (0.5 mL) in the automated headspace was automatically injected into the injection port heated at 250 °C in split mode (10:1). As a carrier gas, helium (purity: 99.999%) was set at a flow rate of 1 mL/min. The oven temperature was programmed to hold at 60 °C for 1 min, ramp up to 200 °C at a rate of 5 °C/min, and then increase to 250 °C at a rate of 10 °C/min. The temperature of the interface, ion source, and quadrupole was set at 250 °C, 230 °C, and 150 °C, respectively, and the EI energy was set at 70 eV. The mass spectra were acquired in scan mode in the range *m*/*z* 40–250 since no substance was detected above *m*/*z* 250 in all *Cannabis* samples in preliminary experiments.

### 3.4. Qualitative and Quantitative Analysis

For qualitative analysis, the individual detection result was compared with the retention time, mass spectral pattern, database of NIST, mass spectra of authentic standards, GC elution order, and *KI* values based on previous reports. *KI* was calculated using a C_8_–C_20_ n-alkane solution and applied to a temperature-programming analysis [36]. Calculated *KI* values were investigated according to the following equation:

(1) $$KI_{x}=100\left(n+\frac{t_{x}-t_{n}}{t_{n+1}-t_{n}}\right)$$

where *n* is the number of n-alkane carbon atoms eluting before the compound *x*; *t_n_* and *t_n_*_+1_ are the retention times that elute before and after compound *x*. The relative abundance of terpenes was individually investigated for each leaf or inflorescence sample of different hybrid *Cannabis* varieties. Quantitative analysis was performed on α- and β-pinene, myrcene, limonene, β-caryophyllene, and α-humulene, known as major terpenes in *Cannabis*. To investigate terpenes and select biomarkers, statistical analyses (such as PCA and HCA) were performed using R 4.1.3 (R Core Team, Vienna, Austria).

### 3.5. Method Validation

For reliable validation, a standard mixture solution of major volatile components in *Cannabis* such as α- and β-pinene, myrcene, limonene, β-caryophyllene, and α-humulene was dissolved in methanol at a concentration of 500 μg/mL using nonane and tetradecane as internal standards. For linearity, standard solutions were prepared at 1–250 μg/mL for α- and β-pinene, limonene, β-caryophyllene, and α-humulene, and 1–500 μg/mL for myrcene. Calibration curves were constructed by comparing the peak area ratios of individual compounds to their internal standards versus their concentrations in μg/mL. LOQ was evaluated as the concentration of a standard mixture with a signal-to-noise ratio (S/N) > 10. To obtain repeatability and reproducibility, intra- and inter-day precision were estimated by analyzing triplicates of *Cannabis* extract. To determine accuracy, *Cannabis* samples were analyzed by spiking the standard solution at three different concentrations (5, 10, and 20 μg/mL). The accuracy data was obtained by calculating differences before and after spiking the standard solution to match the sample matrices.

(2) $$Accuracy=\left(\frac{Amount\;after\;spiking-Amount\;before\;spiking}{spiked\;amount}\right)\times 100\%$$

## 4. Conclusions

In this study, we introduced the automated HS-GC/MS method to simply and easily detect 26 terpenes and quantify six major terpenes, namely α- and β-pinenes, myrcene, limonene, β-caryophyllene, and α-humulene, in the leaves (*n* = 9), and inflorescences (*n* = 7) of hybrid *Cannabis* species without intensive time and labor. To enhance terpene extraction efficiency from leaf and inflorescence samples, the HS incubation time and temperature were optimized at 30 min and 100 °C, respectively. Using the established HS-GC/MS method, 26 terpenes were identified based on EI mass spectral patterns and retention indices. Furthermore, based on the PCA results, we investigated components for identifying individual hybrid *Cannabis* samples and suggested six major terpenes as potential biomarkers. These six terpenes were consistently and predominantly present in all *Cannabis* samples. Furthermore, we quantified six major terpenes in both leaves and inflorescences of hybrid *Cannabis* using HS-GC/MS and tried to demonstrate the “entourage effect” specific to individual *Cannabis* based on quantification results for six terpenes. The HS-GC/MS method used in this study directly detected 26 terpenes and quantified six major terpenes in the leaves and inflorescences of hybrid *Cannabis*. Our research contributes to a better understanding of terpenes’ “entourage effect” in *Cannabis*.

## Figures and Tables

**Table 1:** Retention times, the Kovats index (KI), and characteristic ions of 26 terpenes in hybrid *Cannabis* species.

Elution Order	Compound Name	M.W	RT (Min)	KI calc.	KI Ref	Characteristic Ions *m*/*z* (Relative Abundance%)
1	α-Pinene	136	6.06	935	936	136 (10), 121 (15), 105 (10), 93 (100), 91 (40), 79 (25), 77 (30)
2	β-Pinene	136	7.13	981	978	136 (10), 121 (15), 93 (100), 91 (25), 79 (20), 77 (20), 69 (25)
3	Myrcene	136	7.32	989	989	136 (5), 121 (5), 93 (100), 91 (25), 79 (15), 77 (15), 69 (70), 41 (75)
4	Limonene	136	8.42	1031	1030	136 (25), 121 (25), 107 (25), 93 (75), 79 (35), 68 (100), 67 (70)
5	Eucalyptol	154	8.53	1035	1032	154 (70), 139 (60), 125 (15), 111 (80), 93 (60), 81 (90), 71 (70), 55 (40), 43 (100)
6	*E*-β-Ocimene	136	8.82	1046	1048	136 (5), 121 (20), 105 (20), 93 (100), 91 (45), 80 (35), 79 (40)
7	γ-Terpinene	136	9.19	1060	1060	136 (40), 119 (50), 105 (15), 93 (100), 77 (35), 91 (60)
8	*Z*-Sabinene hydrate	154	9.53	1072	1067	154 (5), 136 (25), 121 (25), 111 (15), 93 (100), 77 (35), 43 (25)
9	β-Caryophyllene	204	19.13	1426	1420	204 (10), 189 (25), 175 (15), 161 (45), 147 (30), 133 (95), 120 (45), 105 (60), 93 (100), 79 (75)
10	trans-α-Bergamotene	204	19.37	1435	1435	204 (5), 189 (5), 161 (5), 119 (100), 107 (30), 93 (95), 79 (25), 69 (35)
11	α-Guaiene	204	19.45	1438	1440	204 (55), 189 (35), 161 (25), 147 (90), 133 (65), 119 (45), 105 (100), 93 (75), 79 (60),
12	*E*-β-Farnesene	204	19.81	1453	1456	204 (5), 189 (5), 161 (15), 133 (30), 120 (25), 107 (10), 93 (65), 79 (25), 69 (100)
13	α-Humulene	204	20.02	1461	1453	204 (10), 189 (5), 161 (5), 147 (20), 121 (40), 107 (15), 93 (100), 80 (30), 67 (10)
14	Alloaromadrene	204	20.12	1465	1460	204 (45), 189 (35), 175 (10), 161 (100), 147 (50), 133 (70), 119 (60), 105 (90), 91 (100)
15	β-Selinene	204	20.85	1494	1486	204 (70), 189 (60), 175 (30), 161 (65), 147 (50), 133 (50), 121 (60), 105 (100), 93 (90)
16	α-Selinene	204	21.01	1500	1493	204 (50), 189 (100),175 (30), 161 (35), 133 (50), 121 (25), 107 (55), 93 (55)
17	*Z,E*-α-Farnesene	204	21.10	1504	1504	204 (5), 161 (10),135 (10), 123 (35), 119 (50), 107 (50), 93 (100), 79 (45), 69 (50)
18	β-Bisabolene	204	21.23	1509	1508	204 (20), 189 (5), 161 (20), 133 (10), 119 (25), 109 (30), 93 (85), 79 (35), 69 (100)
19	β-sesquiphellandrene	204	21.62	1526	1524	204 (30), 189 (5), 161 (60), 133 (40), 120 (30), 109 (30), 93 (70), 69 (100)
20	*E*-α-Bisabolene	204	22.00	1542	1540	204 (20), 189 (5), 161 (5), 147 (5), 136 (10), 119 (30), 109 (25), 93 (100), 78 (25)
21	Selina-3,7(11)-diene	204	22.11	1546	1541	204 (55), 189 (25), 161 (100), 133 (20), 122 (60), 107 (50), 91 (30), 81 (20)
22	Caryophyllene oxide	222	23.10	1587	1581	205 (10), 202 (20), 187 (40), 161 (35), 149 (30), 133 (45), 119 (40), 105 (65), 91 (100), 79 (85)
23	Guaiol	222	23.38	1599	1597	222 (5), 204 (25), 189 (25), 161 (100), 147 (20), 133 (25), 119 (25), 105 (60), 91 (50)
24	γ-Eudesmol	222	24.04	1629	1631	222 (5), 204 (60), 189 (100), 161 (80), 147 (25), 133 (60), 119 (20), 105 (45), 91 (50)
25	Bulnesol	222	24.96	1669	1666	222 (5), 204 (30), 189 (35), 161 (55), 147 (25), 135 (75), 119 (45), 107 (100), 93 (85),
26	α-Bisabolol	222	25.38	1688	1683	204 (30), 189 (5), 161 (20), 135 (10), 119 (90), 109 (95), 93 (85), 79 (40), 69 (100)

**Table 2:** Relative abundances of 26 terpenes in leaves of hybrid *Cannabis*.

Elution Order	Compound Name	Relative Abundance (%)								
		Cherry Blossom	V1	V4	White Widow	Chung Sam	Blue Dream	Bubble Gum	Purple	Victory
1	α-Pinene	28 ± 8	50 ± 30	40 ± 20	2.29 ± 0.04	4.8 ± 0.5	20 ± 10	5 ± 1	38 ± 5	17 ± 1
2	β-Pinene	6.6 ± 0.3	15 ± 3	13 ± 5	1.75 ± 0.09	2.0 ± 0.5	7 ± 5	7 ± 1	14 ± 2	6.5 ± 0.8
3	Myrcene	6.5 ± 0.2	1.14 ± 0.02	2.5 ± 0.2	4.41 ± 0.06	0.08 ± 0.03	10 ± 5	9 ± 2	17 ± 2	14 ± 2
4	Limonene	5 ± 1	2.5 ± 0.6	6.3 ± 0.2	6.5 ± 0.1	1.9 ± 0.6	4 ± 2	19 ± 3	3.4 ± 0.4	3.6 ± 0.6
5	Eucalyptol	0.04± 0.04	1.2 ± 0.9	1.3 ± 0.5	20 ± 5	ND	3 ± 2	ND	ND	0.68 ± 0.02
6	*E*-β-Ocimene	0.6 ± 0.3	0 ± 1	0.47 ± 0.08	ND	ND	1.9 ± 0.6	ND	ND	ND
7	γ-Terpinene	ND	0.1 ± 0.2	0.05 ± 0.07	0.40 ± 0.09	ND	0.6 ± 0.1	ND	0.02 ± 0.01	0.13 ± 0.03
8	*Z*-Sabinene hydrate	ND	0 ± 1	0.3 ± 0.5	0 ± 2	ND	1.0 ± 0.8	ND	0.08 ± 0.05	0.4 ± 0.2
9	β-Caryophyllene	21 ± 3	10 ± 20	10 ± 30	22 ± 5	42 ± 5	20 ± 10	13 ± 3	7 ± 3	18 ± 6
10	trans-α-Bergamotene	4 ± 7	5 ± 9	4 ± 9	1.6 ± 0.5	6.3 ± 0.7	3.7 ± 0.3	5 ± 3	2.6 ± 0.4	5 ± 2
11	α-Guaiene	0.2 ± 0.3	<0.01	ND	0.31 ± 0.01	ND	ND	ND	ND	ND
12	*E*-β-Farnesene	2 ± 4	1 ± 2	0.3 ± 0.7	0.9 ± 0.1	ND	0.62 ± 0.08	0.5 ± 0.5	ND	2 ± 1
13	α-Humulene	16 ± 27	10 ± 20	10 ± 20	16 ± 4	33 ± 4	12.0 ± 0.3	18 ± 8	7.0 ± 0.2	15 ± 2
14	Alloaromadrene	0.2 ± 0.3	0 ± 1	0 ± 1	0.4 ± 0.2	ND	0.3 ± 0.3	ND	0.17 ± 0.09	1.0 ± 0.3
15	β-Selinene	0.3 ± 0.5	ND	ND	1.0 ± 0.4	ND	0.22 ± 0.03	0.57 ± 0.06	0.1 ± 0.1	0.7 ± 0.8
16	α-Selinene	0.3 ± 0.1	ND	ND	1.2 ± 0.3	ND	ND	ND	0.23 ± 0.01	0.90 ± 0.07
17	*Z,E*-α-Farnesene	4 ± 7	0.12 ± 0.07	0.13 ± 0.05	ND	ND	ND	1.4 ± 0.8	2 ± 1	ND
18	β-Bisabolene	4 ± 7	0.3 ± 0.8	0.3 ± 0.8	3.0 ± 0.9	3.4 ± 0.4	1.2 ± 0.6	ND	0.4 ± 0.1	3.8 ± 0.8
19	β-sesquiphellandrene	ND	ND	<0.01	ND	<0.01	ND	ND	ND	0.30 ± 0.07
20	*E*-α-Bisabolene	ND	1 ± 2	1 ± 2	ND	1.1 ± 0.1	ND	9 ± 6	ND	8 ± 2
21	Selina-3,7(11)-diene	ND	ND	ND	17 ± 3	0.6 ± 0.4	5 ± 3	9 ± 3	6.6 ± 0.2	2.7 ± 0.1
22	Caryophyllene oxide	0.43 ± 0.09	1 ± 3	2 ± 6	0.1 ± 0.1	4 ± 1	ND	ND	<0.01	0.02 ± 0.01
23	Guaiol	ND	ND	ND	ND	ND	ND	ND	ND	ND
24	γ-Eudesmol	ND	0 ± 2	0 ± 1	ND	ND	0.2 ± 0.1	ND	ND	ND
25	Bulnesol	ND	0.1 ± 0.3	ND	ND	ND	ND	ND	ND	ND
26	α-Bisabolol	1 ± 3	0.1 ± 0.4	ND	0.8 ± 0.7	1 ± 1	0.2 ± 0.1	ND	0.09 ± 0.01	0.5 ± 0.9

ND means “not detected”.

**Table 3:** Relative abundance of 26 terpenes in the inflorescences of hybrid *Cannabis*.

Elution Order	Compound Name	Relative Abundance (%)						
		Cherry Blossom	V1	V4	White Widow	Chung Sam	Blue Dream	Bubble Gum
1	α-Pinene	29 ± 8	18 ± 7	12 ± 4	3 ± 1	22.8 ± 0.5	20 ± 10	6 ± 1
2	β-Pinene	10 ± 10	10 ± 6	8 ± 4	5 ± 3	3.8 ± 0.4	9 ± 3	10 ± 2
3	Myrcene	40 ± 30	50 ± 20	2 ± 1	40 ± 10	0.13 ± 0.02	41 ± 5	16 ± 2
4	Limonene	6 ± 3	6.6 ± 0.9	10 ± 6	13 ± 9	0.2 ± 0.2	2.3 ± 0.6	35 ± 1
5	Eucalyptol	ND	0.06 ± 0.08	ND	0.5 ± 0.4	ND	ND	<0.01
6	*E*-β-Ocimene	5 ± 2	9.9 ± 0.5	0.9 ± 0.7	3 ± 1	ND	9 ± 2	ND
7	γ-Terpinene	0.0 ± 0.1	0.07 ± 0.03	1.1 ± 0.8	0.19 ± 0.02	0.06 ± 0.03	0.17 ± 0.01	0.07 ± 0.05
8	*Z*-Sabinene hydrate	0.02 ± 0.01	0.08 ± 0.01	0.2 ± 0.2	0.11 ± 0.06	<0.01	0.09 ± 0.01	ND
9	β-Caryophyllene	3 ± 1	0.7 ± 0.5	20 ± 10	14.0 ± 0.9	31 ± 4	6 ± 4	10.2 ± 0.3
10	trans-α-Bergamotene	0.9 ± 0.4	0.09 ± 0.06	6.4 ± 0.9	0.4 ± 0.2	8 ± 5	0.66 ± 0.03	0.47 ± 0.03
11	α-Guaiene	0.21 ± 0.09	0.19 ± 0.03	0.0 ± 0.2	2.4 ± 0.3	0.9 ± 0.1	<0.01	<0.01
12	*E*-β-Farnesene	1.3 ± 0.6	0.05 ± 0.03	0.77 ± 0.09	0.5 ± 0.3	0.22 ± 0.04	0.08 ± 0.01	0.12 ± 0.09
13	α-Humulene	2.1 ± 0.9	0.5 ± 0.5	21 ± 2	11 ± 2	24 ± 4	4 ± 2	7.4 ± 0.1
14	Alloaromadrene	0.13 ± 0.05	<0.01	0.4 ± 0.2	0.11 ± 0.04	ND	0.09 ± 0.01	ND
15	β-Selinene	0.04 ± 0.02	0.03 ± 0.01	0.3 ± 0.2	0.8 ± 0.2	1 ± 1	0.20 ± 0.03	0.75 ± 0.07
16	α-Selinene	0.04 ± 0.01	0.04 ± 0.09	0.29 ± 0.06	1 ± 1	1.5 ± 0.2	0.22 ± 0.02	0.9 ± 0.4
17	*Z,E*-α-Farnesene	0.38 ± 0.09	0.2 ± 0.3	2.6 ± 0.3	2.0 ± 0.6	1.40 ± 0.06	0.13 ± 0.03	0.7 ± 0.1
18	β-Bisabolene	0.5 ± 0.2	0.02 ± 0.01	2.9 ± 0.4	0 ± 1	0.3 ± 0.3	0.06 ± 0.02	0.08 ± 0.05
19	β-sesquiphellandrene	0.09 ± 0.04	ND	ND	0.1 ± 0.1	ND	0.05 ± 0.03	ND
20	*E*-α-Bisabolene	0.6 ± 0.3	0.0 ± 0.2	3.9 ± 0.5	2 ± 2	4 ± 3	1.7 ± 0.5	ND
21	Selina-3,7(11)-diene	ND	ND	0.3 ± 0.1	3 ± 2	0.1 ± 0.5	1.5 ± 0.3	12 ± 1
22	Caryophyllene oxide	<0.01	0.0 ± 0.1	0.7 ± 0.4	0.06 ± 0.01	0.2 ± 0.2	ND	ND
23	Guaiol	0.03 ± 0.02	0.1 ± 0.2	0.3 ± 0.1	ND	ND	0.12 ± 0.08	ND
24	γ-Eudesmol	0.05 ± 0.03	0.05 ± 0.01	0.5 ± 0.3	ND	ND	0.2 ± 0.2	ND
25	Bulnesol	ND	ND	ND	ND	ND	0.07 ± 0.05	ND
26	α-Bisabolol	ND	ND	0.4 ± 0.7	0.15 ± 0.06	0.03 ± 0.01	ND	ND

ND means “not detected”.

**Table 4:** Quantification results for six major terpenes in the leaf and inflorescence samples of hybrid *Cannabis*.

Strains	Organ	Concentrations (μg/g)					
		α-Pinene	β-Pinene	Myrcene	Limonene	β-Caryophyllene	α-Humulene
Cherry Blossom	leaf	144 ± 8	32.9 ± 0.5	187 ± 7	14 ± 1	1200 ± 600	300 ± 100
	inflorescence	2270 ± 70	930 ± 20	17,400 ± 300	260 ± 20	2000 ± 100	500 ± 30
V1	leaf	100 ± 10	80 ± 60	10 ± 10	8 ± 6	220 ± 20	66 ± 4
	inflorescence	500 ± 100	290 ± 60	4600 ± 400	115 ± 9	200 ± 10	40 ± 30
V4	leaf	84 ± 5	27 ± 3	20 ± 10	8 ± 2	240 ± 60	70 ± 20
	inflorescence	93 ± 1	60 ± 30	50 ± 50	47 ± 4	2500 ± 300	690 ± 80
White Widow	leaf	9.8 ± 0.6	6.2 ± 0.4	79 ± 7	13 ± 1	950 ± 10	236 ± 5
	inflorescence	64 ± 3	110 ± 10	2000 ± 1000	150 ± 30	3300 ± 400	870 ± 80
Chung Sam	leaf	6.9 ± 0.3	2.6 ± 0.2	0.7 ± 0.2	1.6 ± 0.2	263 ± 4	73 ± 1
	inflorescence	183 ± 5	30.1 ± 0.3	6.1 ± 0.1	1.3 ± 0.2	2900 ± 600	800 ± 100
Blue Dream	leaf	56.5 ± 0.7	18.6 ± 0.6	200 ± 100	6.1 ± 0.1	205 ± 4	51 ± 4
	inflorescence	200 ± 300	116 ± 3	1400 ± 900	19 ± 1	400 ± 200	100 ± 60
Bubble Gum	leaf	9.79 ± 0.02	10.6 ± 0.2	75 ± 1	16.2 ± 0.6	60 ± 40	48 ± 2
	inflorescence	93.17 ± 0.03	142 ± 1	1310 ± 30	300 ± 20	500 ± 100	130 ± 40
Purple	leaf	450 ± 10	200 ± 50	1190 ± 60	34 ± 6	400 ± 70	130 ± 30
Victory	leaf	332 ± 3	122 ± 4	1550 ± 60	42 ± 3	1100 ± 200	300 ± 70

## Data Availability

Data are available from a publicly accessible repository.

## Supplementary Materials

The following supporting information can be downloaded at: https://www.mdpi.com/article/10.3390/molecules28248082/s1, Figure S1: Total ion chromatograms of terpenes in leaves of Victory at different incubation temperature of (a) 60 °C, (b) 80 °C, (c) 100 °C, and (d) 120 °C; Figure S2: Cluster dendrogram of leaves and inflorescences of hybrid *Cannabis* using relative abundances of peak areas for 26 terpenes; Table S1: Analytical characteristics of the HS-GC/MS method for 6 major terpenes.
