# Supplementary material for: Identification of Terpene Compositions in the Leaves and Inflorescences of Hybrid Cannabis Species Using Headspace-Gas Chromatography/Mass Spectrometry
Source: Molecules. 2023 Dec 14;28(24):8082. doi: 10.3390/molecules28248082 (PMC10745826; doi:10.3390/molecules28248082)
Supplement: Supplementary file 1 [file molecules-28-08082-s001.zip › molecules-2705124-supplementary.pdf]

Supplementary Materials

Identification of Terpene Compositions in the Leaves and Inflorescences of Cybrid *Cannabis* Species Using Headspace-Gas Chromatography/Mass Spectrometry

Sangin Lee[a], Eun Jae Kim[a], Eunjeong Kwon[a], Seo Jeong Oh[a], Mansoo Cho[b], Chul Min Kim[c], Wonwoong Lee[d,*], Jongki Hong[a,*]

[a]College of Pharmacy, Kyung Hee University, Seoul 02447, Republic of Korea

[b]Graduate school of Techno Design, Kookmin University, Seoul 02707, Republic of Korea

[c]Department of Horticulture Industry, Wonkwang University, Iksan 54538, Republic of Korea

[d]Research Institute of Pharmaceutical Sciences, College of Pharmacy, Woosuk University, Wanju 55338, Republic of Korea

[*] Corresponding author: Tel: +82−2−961−9255; Fax: +82−2−961–0357;

E-mail: jhong@khu.ac.kr

[*] Co-corresponding author: Tel: +82−63−290−1573;

E-mail: wwlee@woosuk.ac.kr

Figure S1. Total ion chromatograms of terpenes in leaves of Victory at different incubation temperature of (a) 60°C, (b) 80°C, (c) 100°C, and (d) 120°C.

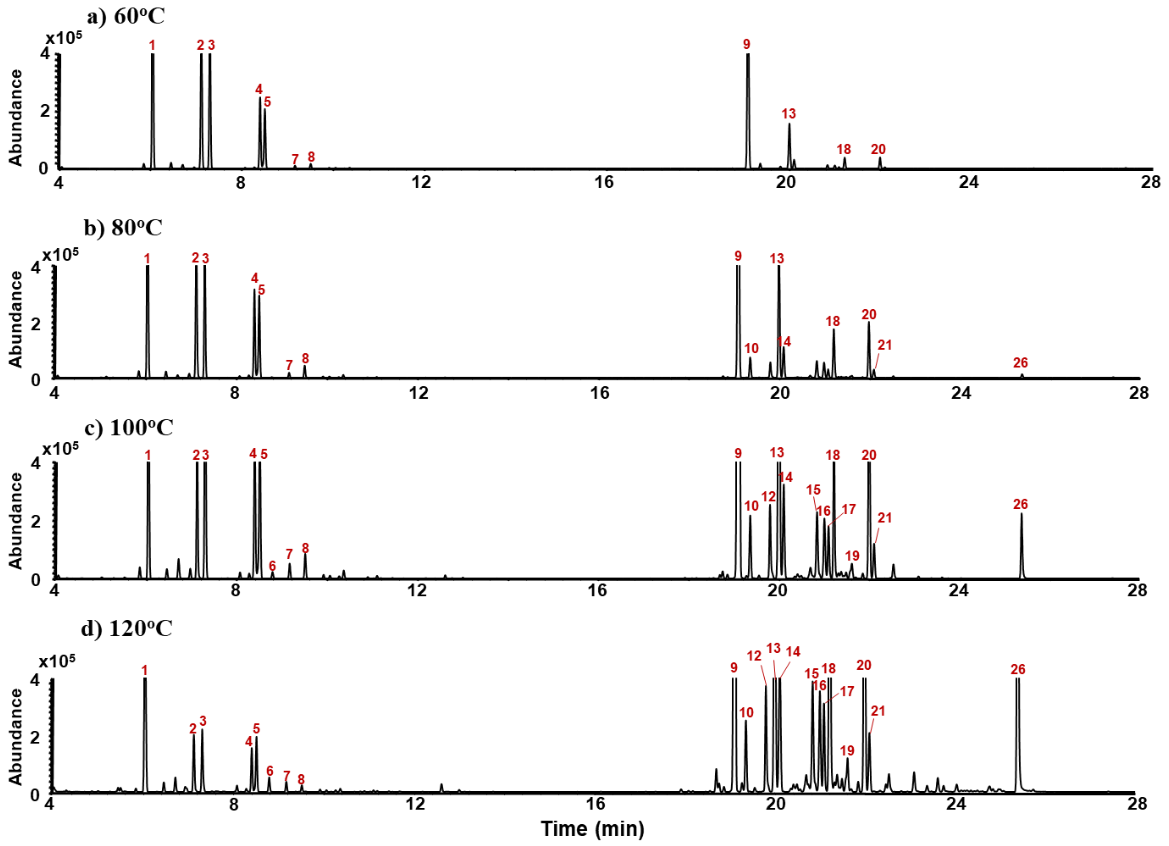

Figure S2. Cluster dendrogram of leaves and inflorescences of hybrid *Cannabis* using relative abundances of peak areas for 26 terpenes.

Numbers were identified as follows: 1. Cherry Blossom leaf, 2. V1 leaf, 3. V4 leaf, 4. White Widow leaf, 5. Chung Sam leaf, 6. Blue Dream leaf, 7. Bubble Gum leaf, 8. Purple leaf, 9. Victory leaf, 10. Cherry Blossom inflorescence, 11. V1 inflorescence, 12. V4 inflorescence, 13. White Widow inflorescence, 14. Chung Sam inflorescence, 15. Blue Dream inflorescence, 16. Bubble Gum inflorescence.

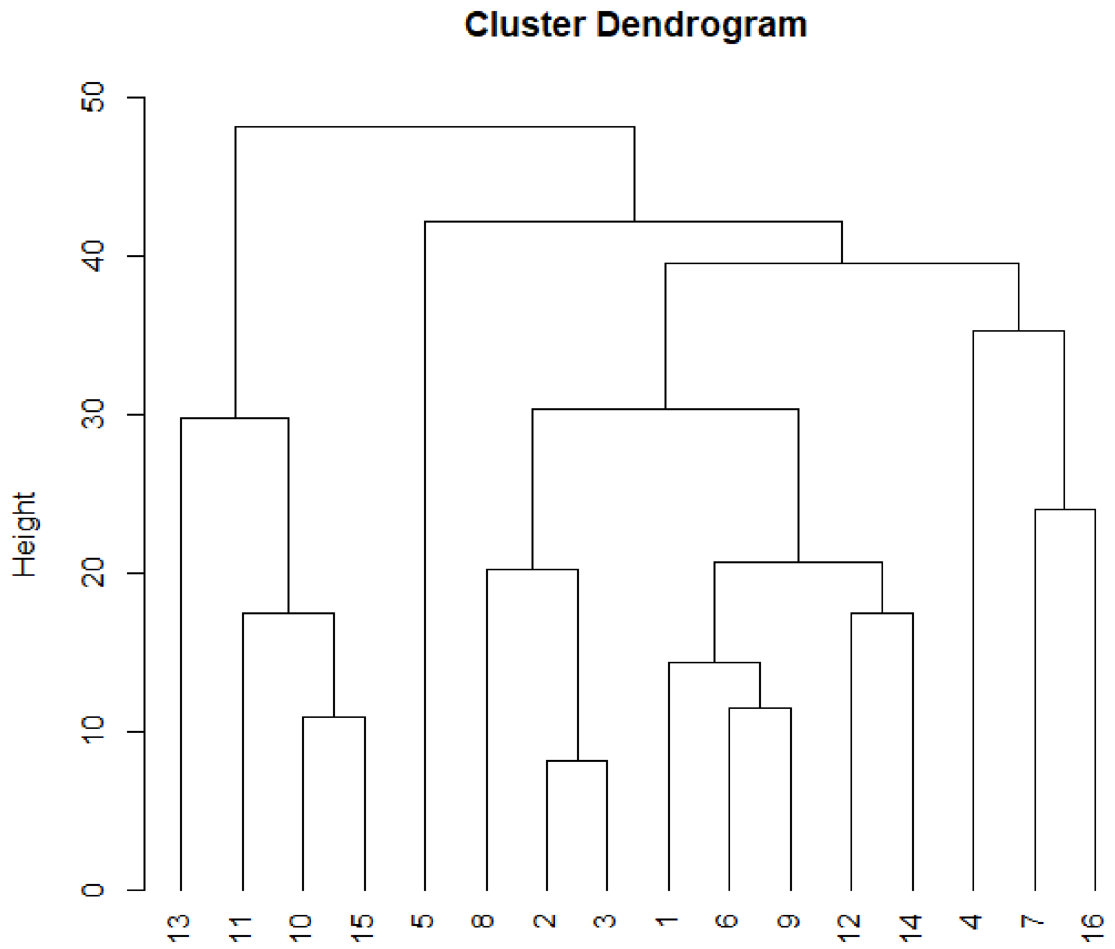

Table S1. Analytical characteristics of the HS-GC/MS method for 6 major terpenes.

Terpenes	Extract ion (m/z)	LOQ (ng/mL)	Linearity		Precision		Accuracy		
			Range (μg/mL)	r^2	Intra-day (%)	Inter-day (%)	5 μg/mL (%)	10 μg/mL (%)	20 μg/mL (%)
α-Pinene	93	10	1 - 250	0.9954	3.02±0.91	6.48±1.21	102.01±0.533	103.41±2.212	98.98±2.534
β-Pinene	93	10	1-250	0.9956	3.97±1.021	5.78±0.854	100.74±2.95	101.52±1.51	104.92±1.36
Myrcene	93	100	1 – 500	0.9986	4.47±1.59	6.55±1.64	82.66±0.62	92.25±2.705	96.00±1.521
Limonene	68	10	1-250	0.9976	2.23±0.49	4.48±1.42	102.10±2.47	102.39±2.16	103.84±2.96
β-Caryophyllene	93	10	1 – 250	0.9976	8.01±1.40	10.19±1.06	108.66±2.86	111.56±12.054	108.75±3.15
α-Humulene	93	10	1 - 250	0.9961	2.90±0.89	3.29±1.13	86.31±1.774	94.87±2.82	104.10±8.297

All precision and accuracy data were obtained by analysis in triplicate.
